# Phenomics and Genomics Reveal Adaptation of *Virgibacillus dokdonensis* Strain 21D to Its Origin of Isolation, the Seawater-Brine Interface of the Mediterranean Sea Deep Hypersaline Anoxic Basin Discovery

**DOI:** 10.3389/fmicb.2019.01304

**Published:** 2019-06-12

**Authors:** Zahraa Zeaiter, Ramona Marasco, Jenny M. Booth, Erica M. Prosdocimi, Francesca Mapelli, Matteo Callegari, Marco Fusi, Grégoire Michoud, Francesco Molinari, Daniele Daffonchio, Sara Borin, Elena Crotti

**Affiliations:** ^1^Dipartimento di Scienze per gli Alimenti, la Nutrizione e l’Ambiente (DeFENS), Università degli Studi di Milano, Milan, Italy; ^2^Red Sea Research Center (RSRC), King Abdullah University of Science and Technology (KAUST), Thuwal, Saudi Arabia

**Keywords:** brine pools, osmoadaptation, salt stress, magnesium chloride, spore

## Abstract

The adaptation of sporeformers to extreme environmental conditions is frequently questioned due to their capacity to produce highly resistant endospores that are considered as resting contaminants, not representing populations adapted to the system. In this work, in order to gain a better understanding of bacterial adaptation to extreme habitats, we investigated the phenotypic and genomic characteristics of the halophile *Virgibacillus* sp. 21D isolated from the seawater-brine interface (SBI) of the MgCl_2_-saturated deep hypersaline anoxic basin Discovery located in the Eastern Mediterranean Sea. Vegetative cells of strain 21D showed the ability to grow in the presence of high concentrations of MgCl_2,_ such as 14.28% corresponding to 1.5 M. Biolog phenotype MicroArray (PM) was adopted to investigate the strain phenotype, with reference to carbon energy utilization and osmotic tolerance. The strain was able to metabolize only 8.4% of 190 carbon sources provided in the PM1 and PM2 plates, mainly carbohydrates, in accordance with the low availability of nutrients in its habitat of origin. By using *in silico* DNA-DNA hybridization the analysis of strain 21D genome, assembled in one circular contig, revealed that the strain belongs to the species *Virgibacillus dokdonensis*. The genome presented compatible solute-based osmoadaptation traits, including genes encoding for osmotically activated glycine-betaine/carnitine/choline ABC transporters, as well as ectoine synthase enzymes. Osmoadaptation of the strain was then confirmed with phenotypic assays by using the osmolyte PM9 Biolog plate and growth experiments. Furthermore, the neutral isoelectric point of the reconstructed proteome suggested that the strain osmoadaptation was mainly mediated by compatible solutes. The presence of genes involved in iron acquisition and metabolism indicated that osmoadaptation was tailored to the iron-depleted saline waters of the Discovery SBI. Overall, both phenomics and genomics highlighted the potential capability of *V. dokdonensis* 21D vegetative cells to adapt to the environmental conditions in Discovery SBI.

## Introduction

Microorganisms inhabit all the habitats present on Earth, including those environments indicated as “extreme” and characterized by strong selective physico-chemical forces, e.g., polar seas, cold and hot deserts, and hydrothermal vents. These extreme environments represent a fascinating source of bacterial diversity and metabolic activities with interesting biotechnological potential (De Vitis et al., 2015; Raddadi et al., 2015, 2018).

Microorganisms living in extreme habitats, namely “extremophiles”, show metabolic and physiological adaptations to their environmental conditions. For instance, they can possess atypical enzymes that are adapted to the extreme conditions (Mapelli et al., 2012; Gunde-Cimerman et al., 2018). The enzymatic adaptation to high-osmolarity stress, included in the so-called “salt-in” strategy, is typical of those microorganisms that adjust their cell turgor pressure in order to avoid any water loss. Examples include the extremely halophilic strains of the Euryarchaeota order of Halobacteriales and the Bacteroidetes bacterium *Salinibacter ruber*. Conversely, microbial cells can respond to the high-osmolarity stress using a different strategy, known as “low-salt-in” or “compatible solute” strategy, which consists of the intracellular accumulation or uptake of compatible solutes from the surrounding environment in order to balance the cytoplasmic content with the outside (Oren, 2008).

It is generally thought that when unfavorable conditions are present in a specific environment, the inhabiting or transient microbial cells can enter the state of “dormancy” in order to thrive once the stressful conditions are overcome (Lennon and Jones, 2011). Harsh conditions that encourage dormancy include lack of resources, high residence time, presence of predators, occurrence of a perturbation regime and stresses, such as high temperature, desiccation, high UV irradiation, and chemical damage (Lennon and Jones, 2011). Endospores are one of the dormancy expressions, fundamental for the persistence of a certain cell population or genotype with consequent important repercussions at the community and ecosystem levels (Lennon and Jones, 2011). Nevertheless, spore production represents a costly involvement for sporeformers, since it involves not only bacterial genome replication, but also the biosynthesis of cell structures, e.g., protective layers, as well as the death of the mother cell. Moreover, authors have described that under high salinity stress *Bacillus subtilis* cells can block the entry into the sporulation pathway in order to prevent the osmotically damaged cells committing to a developmental program that they cannot complete (Kunst and Rapoport, 1995; Ruzal et al., 1998; Widderich et al., 2016).

Deep hypersaline anoxic basins (DHABs) have attracted particular interest from researchers and several basins have been discovered in the Eastern Mediterranean Sea, e.g., L’Atalante, Bannock, Discovery, Urania, Thetis, and Kryos (Mapelli et al., 2016; Barozzi et al., 2018; Merlino et al., 2018). These basins are highly saline lakes located on the seafloor, at around 3000 m below sea level, characterized by anoxia, high hydrostatic pressure and a sharp chemocline at the seawater-brine interface (SBI) (van der Wielen et al., 2005; Daffonchio et al., 2006; Yakimov et al., 2007; Borin et al., 2009). Each brine pool has a peculiar chemical composition that selects specialized prokaryotic communities (van der Wielen et al., 2005). For example, the Discovery basin is characterized by the presence of a high concentration of MgCl_2_ (5 M) and it has been indicated as one of the most extreme environments on Earth (Hallsworth et al., 2007; Lee et al., 2018; Steinle et al., 2018; La Cono et al., 2019). Discovery basin was formed 2000 years ago by the dissolution of evaporates made of bischofite [(MgCl_2_)⋅H_2_O] (Wallmann et al., 1997) and its SBI is characterized by a steep MgCl_2_ gradient ranging from the value of seawater, i.e., 0.48% (0.05 M), to that of the brine, i.e., 48.08% (5.05 M) (Hallsworth et al., 2007). MgCl_2_ is a remarkably chaotropic salt, able to reduce the enzymatic activity of glucose-6-phosphate dehydrogenase in laboratory conditions by 80 and 95% when present at concentrations of 1.90% (0.2 M) and 3.81% (0.4 M), respectively (Hallsworth et al., 2007). Experiments of enzymatic inactivation, performed using samples from Discovery brine and SBI instead of MgCl_2_ pure solutions, also displayed similar results (Hallsworth et al., 2007). Due to the chaotropicity of MgCl_2_ with the absence of other compensating ions, the high MgCl_2_ concentration has been suggested to create conditions unsuitable for life (Hallsworth et al., 2007), although the recent study on the microbial community thriving in Kryos brine pool extended the chaotropicity limit of life (Yakimov et al., 2015). Furthermore, although DHABs are generally regarded as aggressive environments for cells and macromolecules, naked DNA has been shown to be preserved in this habitat, maintaining its transforming potential (Borin et al., 2008; Yakimov et al., 2015).

The adaptation of bacteria able to produce endospores, i.e., sporeformers, to extreme environmental conditions is frequently questioned, since highly resistant spores are generally considered as resting contaminants, not representing populations adapted to the ecosystem. Here, in order to verify this assumption, we investigated the phenotypic and genomic characteristics of *Virgibacillus* sp. strain 21D, isolated from the SBI of the DHAB Discovery located in the Eastern Mediterranean Sea (De Vitis et al., 2015).

## Materials and Methods

### Bacterial Strain and Cultivation Media

*Virgibacillus* sp. strain 21D was isolated on 246 DSM medium from a SBI sample collected from the Discovery (35°17′ N, 21°41′ E) DHAB (De Vitis et al., 2015). Briefly, serial dilutions of the Discovery interface (1 ml) sample were plated on 246 DSM medium added with cycloheximide 100 μg/ml and incubated at 30°C until the appearance of bacterial colonies. One colony, streaked three times to ensure the purity, was labeled with name 21D and subjected to genotypic characterization and identification, as described by De Vitis et al. (2015). Strain 21D is routinely maintained on marine broth (MB) medium (Conda) by incubation for 48 h at 30°C. Gram staining was performed following the standard Gram procedure. Growth optimum at different NaCl (0, 3, 6, 9, and 12%) and MgCl_2_ (0, 0.95, 4.76, 9.52, 11.43, 14.28, and 17.14%) concentrations was tested on Plate Count Broth (PCB; supplemented with 6% NaCl in case of the growing tests with MgCl_2_) by using 96-well microplate. Bacterial growth in 96-well microplate at 30°C was monitored at 610 nm by using a microplate reader (TECAN Infinite Pro200). Elaboration of the retrieved data was performed using Excel software. MgCl_2_ percentage at which the growth curve showed the higher slope value was considered as optimum MgCl_2_ concentration. Similarly, the growth of strain 21D in the presence of different osmolytes, i.e., betaine (0.5, 1, 2, 5, and 10%), choline (0.5, 1, 2, 5, and 10%), ethylene glycol (5, 10, 20, and 50%), and potassium chloride (3, 6, 9, and 12%), was monitored in 96-well microplates at 30°C in PCB supplemented with 6% NaCl by using a microplate reader (TECAN Infinite Pro200). Chitinolytic ability of the strain was determined by growth on MB agar plates supplemented with 7% colloidal chitin for 10 days at 30°C (Raddadi et al., 2009).

### Scanning Electron Microscopic Analysis

Cells of *Virgibacillus* sp. strain 21D were filtered onto 0.1 μm polycarbonate Whatman filters (Nucleopore) before fixation with 2.5% glutaraldehyde in 0.1 M cacodylate buffer. Filters were washed in 0.1 M cacodylate buffer and subsequently post-fixed in osmium tetroxide. Samples were washed with an ethanol gradient (20–100% ethanol) and subject to critical point drying (Autosamdri-815B, Tousimis). Filters were coated with a 5 nm layer of Au/Pb using a K575X sputter coater (Quorum) and images were acquired using a Quanta 600 FEG (Thermo Scientific) scanning electron microscope at an acceleration voltage of 5 kV.

### Biolog Phenotype MicroArray (PM)

All the materials and reagents used were purchased from Biolog (Hayward, CA, United States). Plates PM1-2, assessing the phenotypes of different carbon sources, and plate PM9, testing for osmotic/ion and pH effects, were used. Strain 21D isolate was streaked on MB agar plates and incubated at 30°C in darkness for 48 h. Cells were scraped from the surface of the plates and inoculated in 20 ml 0.9% NaCl to reach 81–85% transmittance of the cell suspension. For PM1 and PM2, 1.76 ml of cell suspension were added to 20 ml of M9 medium without C sources (12.8 g/L Na_2_HPO_4_, 3 g/L KH_2_PO_4_, 0.5 g/L NaCl, 1 g/L NH_4_Cl, 0.24 g/L MgSO_4_, 0.01 g/L di CaCl_2_) added with 5% NaCl, together with 0.24 ml of Dye F and 2 ml of PM1-2 additive solution. PM9 was inoculated with an inoculating fluid composed by 10 ml of IF-10b GN/GP, 0.88 ml of cell suspension, 0.12 ml of Dye F and 1 ml of PM additive solution. All the plates were run in duplicates and incubated in the OmniLog incubator for 96 h (PM1–2) or 72 h (PM9) at 30°C. Data was collected every 15 min by the OmniLog incubator and analyzed using Kinetic and Parametric software (Biolog). Phenotype responses were analyzed observing the presence of area under the kinetic curve or reporting the highest point reached by the strain growth curve (PM9). Assays in well A01 for PM1 and PM2 was a negative control.

### DNA Preparation

Genomic DNA was prepared from an overnight culture of *Virgibacillus* sp. strain 21D in MB medium at 30°C (150 rpm) using DNeasy Blood & Tissue Kit (Qiagen, Italy) following the manufacturer’s protocol for Gram-positive bacteria. Quantification and quality control of the DNA was performed by spectrophotometry and agarose (0.8%) gel electrophoresis.

### Whole Genome Sequencing, Assembly, and Annotation

The genome of *Virgibacillus* sp. strain 21D was sequenced using PacBio (Pacific Biosciences, CA, United States) technology at Macrogen (South Korea). Specifically, a library with size of 10 kbp was prepared and 1 SMRT cell used for sequencing. Raw reads were filtered and *de novo* assembled using FALCON Assembler software (v0.2.2 release, January 2016) (Koren and Phillippy, 2015). After whole genome assembly, the complete genome of *Virgibacillus* sp. strain 21D was annotated using the pipeline Prokka (Seemann, 2014). The complete genome sequence was deposited in GenBank under the accession number CP018622, BioProject PRJNA354837, BioSample SAMN06052362.

### Taxonomic Position of Strain 21D

We used the GTDB-Tk software^1^ to define the taxonomic position of the strain 21D in the newly described Genome Taxonomy Database (GTDB, Parks et al., 2018). The analysis was performed by concatenating 120 single copy genes present in the reference genomes (accession numbers are reported in Table 1), and in the genome of strain 21D.

**Table 1:** Accession numbers of genomes used in the GTDB analysis.

NCBI Taxonomy	Accession Number
*Bacillus megaterium* MSP20.1	RS_GCF_000480335.1
*Virgibacillus alimentarius* J18T	RS_GCF_000709085.1
*Virgibacillus massiliensis* Vm-5	RS_GCF_000723585.1
*Virgibacillus* sp. SK37	RS_GCF_000725285.1
*Virgibacillus pantothenticus* DSM 26	RS_GCF_001189575.1
*Virgibacillus halodenitrificans* JCM 12304	RS_GCF_001310895.1
*Bacillus niameyensis* SIT3	RS_GCF_001375535.1
*Oceanobacillus picturae* Heshi-B3	RS_GCF_001485235.1
*Virgibacillus* sp. LM2416	RS_GCF_002216775.1
*Virgibacillus necropolis* LMG 19488	RS_GCF_002224365.1
*Virgibacillus salinus* CGMCC 1.10449	RS_GCF_900102415.1
*Virgibacillus subterraneus* CGMCC 1.7734	RS_GCF_900110695.1
*Virgibacillus chiguensis* CGMCC 1.6496	RS_GCF_900129865.1
*Virgibacillus dakarensis* Marseille-P3469	RS_GCF_900155625.1
*Virgibacillus proomii* V-P	RS_GCF_900162615.1
*Virgibacillus siamensis* Marseille-P2607	RS_GCF_900162695.1
*Virgibacillus dokdonensis* Marseille-P2545	RS_GCF_900166595.1
*Virgibacillus ndiopensis* Marseille-P3835	RS_GCF_900187325.1

*In silico* DNA–DNA hybridization (digital DDH, dDDH) was computed using the recommended settings of the Genome-to-Genome Distance Calculator (GGDC) web server version 2.1 (Meier-Kolthoff et al., 2013, 2014) available at the website http://ggdc.dsmz.de/. Closest relative species and bacterial species included in *Virgibacillus* genus were considered.

### Determination of the Proteome Isoelectric Point

Genome sequence of *Virgibacillus* sp. strain 21D was used to infer the isoelectric point (pI) of the proteome by means of the expasy server (Gasteiger et al., 2005). The pI of strains *Escherichia coli* DSM 30083, *V. pantothenticus* DSM 26^T^, *V. chiguensis* NTU-101^T^ and *V. dokdonensis* Marseille-P2545, *V. halodenitrificans* JCM 12304, *S. ruber* DSM 13855, *Desulfohalobium retbaense* DSM 5692 were also computed for comparison purposes.

## Results

### Phenotypic Characterization

**FIGURE 1:**
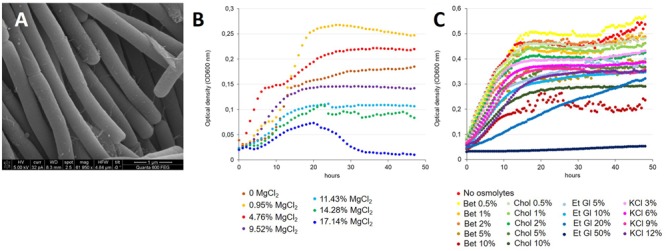
Morphology **(A)** and growth **(B,C)** of *V. dokdonensis* strain 21D. In **(A)**, SEM image of strain 21D. In **(B)**, strain 21D growth in presence of increasing concentrations of MgCl_2_. In **(C)**, strain 21D growth in presence of increasing concentrations of different osmolytes. Bet, betaine; Chol, choline; Et Gl, ethylene glycol; KCl.

*Virgibacillus* sp. strain 21D is a Gram-variable, motile, and spore-forming bacterium with rod shaped cells (Figure 1A and Supplementary Figure 1). When grown on MB agar plates at 30°C in aerobic conditions, it forms 1–2 mm colonies within 48 h. The isolate can grow at 25, 30, 37, 42, and 50°C. The cells show an average width of 0.4 μm and length of 1.5 μm, as confirmed by scanning electron microscopy (Figure 1A; Heyrman et al., 2003). We observed the capability of cells to form long filamentous chains (Figure 1A), consistent with previous observations of *Virgibacillus* spp. strains (Yoon et al., 2005; Wang et al., 2008, 2015). Growth was observed in (i) PCB medium containing NaCl in concentrations ranging from 3 to 10% with optimal growth value between 6 and 10% (also considering Biolog experiments); and (ii) in PCB (added with 6% NaCl) containing MgCl_2_ in concentrations ranging between 0 and 17.14% (Figure 1B). Specifically, the strain 21D was able to grow at high concentrations of MgCl_2_, i.e., 11.43 and 14.28%, which corresponded to 1.2 and 1.5 M MgCl_2_. Even in the presence of 17.14% (1.8 M) MgCl_2_, we verified low growth of the strain 21D, which peaks after 20 h of incubation, followed by a sharp decrease of the optical density (OD) (Figure 1B). Low concentrations of MgCl_2_, i.e., 0.95 and 4.76% (0.1 and 0.5 M), are required to reach high OD values if compared with 0% MgCl_2_ growth (Figure 1B).

Strain 21D was also grown in the presence of different concentrations of the following osmolytes: betaine, choline, ethylene glycol and KCl (Figure 1C). In PCB 6%NaCl, strain 21D showed the ability to grow similarly to the positive control when 0.5, 1, or 2% betaine was added to the medium, whereas at concentrations of 5 and 10% betaine a slight decrease in OD values (more marked with 10% betaine) occurred. A similar trend was found in the presence of choline, whereas KCl addition did allow the strain to grow well, although at lower OD values than in the absence of osmolytes in the growth medium (line in red in Figure 1C). In presence of 20 and 50% ethylene glycol, a reduced growth and a strong inhibition of strain 21D were found respectively, while the addition of 5 and 10% of the osmolyte did slightly affect the strain growth.

Vegetative cells of strain 21D were able to metabolize 8.4% of the tested carbon sources (8 out of 95 in plate PM1 and 8 out of 95 in plate PM2) (Table 2). They showed the capability to metabolize glucose, ribose, arbutin, tagatose, and glucosamine, while they were not able to hydrolyse chitin in the adopted experimental conditions. Plate PM9 was used to test the bacterial metabolic activity in presence of different stress conditions. We confirmed the strain activity in presence of NaCl (with the optimal activity between 6 and 10% NaCl), as well as in presence of different osmolytes and ions (Table 3 and Supplementary Figure 2). We detected a reduction of the bacterial metabolism in certain cases, i.e., in presence of 1–4% NaCl, 2% sodium sulfate, 5–20% ethylene glycol, 1–2% sodium formate, 2% urea, 10–100 mM ammonium sulfate (pH 8.0) and 10–100 mmol/L sodium nitrate. A strong reduction was also detected with 3–7% urea, 20–200 mM sodium benzoate (pH 5.2) and 10–100 mM sodium nitrite (Supplementary Figure 2).

**Table 2:** Substrates in Biolog PM1 and PM2 MicroPlates metabolized by *V. dokdonensis* strain 21D.

Plate PM1		Plate PM2	
Well	Chemical	Well	Chemical
A11	D-Mannose	A06	Dextrin
B03	Glycerol	A10	Laminarin
C04	D-Ribose	A12	Pectin
C07	D-Fructose	B08	Arbutin
C09	a-D-Glucose	D06	D-Tagatose
C10	Maltose	E05	D-Glucosamine
E10	Maltotriose	F05	Oxalomalic acid
F03	*m*-Inositol	H12	3-Hydroxy-2-butanone

**Table 3:** Metabolic profiling of *V. dokdonensis* strain 21D on Biolog PM 9 MicroPlate.

Well	Substrate	21D	Well	Substrate	21D	Well	Substrate	21D	Well	Substrate	21D
A1	NaCl 1 %	+	C1	NaCl 6 % + KCl	++	E1	Sodium formate 1%	+	G1	Sodium Phosphate pH 7 20 mM	+
A2	NaCl 2 %	+	C2	NaCl 6 % + L-proline	++	E2	Sodium formate 2%	+	G2	Sodium Phosphate pH 7 50 mM	+
A3	NaCl 3 %	+	C3	NaCl 6 % + N-Acethyl L-glutamine	++	E3	Sodium formate 3%	++	G3	Sodium Phosphate pH 7 100 mM	+
A4	NaCl 4 %	+	C4	NaCl 6 % + ß-Glutamic acid	++	E4	Sodium formate 4%	++	G4	Sodium Phosphate pH 7 200 mM	+
A5	NaCl 5 %	++	C5	NaCl 6 % + γ-Amino-n-butyric acid	++	E5	Sodium formate 5%	++	G5	Sodium Benzoate pH 5.2 20 mM	–
A6	NaCl 5.5 %	++	C6	NaCl 6 % + Glutathione	++	E6	Sodium formate 6%	++	G6	Sodium Benzoate pH 5.2 50 mM	–
A7	NaCl 6 %	++	C7	NaCl 6 % + Glycerol	++	E7	Urea 2%	+	G7	Sodium Benzoate pH 5.2 100 mM	–
A8	NaCl 6.5 %	++	C8	NaCl 6 % + Trehalose	++	E8	Urea 3%	–	G8	Sodium Benzoate pH 5.2 200 mM	+
A9	NaCl 7 %	++	C9	NaCl 6 % + Trimethylamine-N-oxide	++	E9	Urea 4%	–	G9	Ammonium sulfate pH 8 10 mM	+
A10	NaCl 8 %	++	C10	NaCl 6 % + Trimethylamine	++	E10	Urea 5%	–	G10	Ammonium sulfate pH 8 20 mM	+
A11	NaCl 9 %	++	C11	NaCl 6 % + Octopine	++	E11	Urea 6%	–	G11	Ammonium sulfate pH 8 50 mM	–
A12	NaCl 10 %	++	C12	NaCl 6 % + Trigonelline	++	E12	Urea 7%	–	G12	Ammonium sulfate pH 8 100 mM	+
B1	NaCl 6 %	++	D1	Potassium chloride 3%	++	F1	Sodium Lactate 1%	++	H1	Sodium Nitrate 10 mM	+
B2	NaCl 6 % + Betaine	++	D2	Potassium chloride 4%	++	F2	Sodium Lactate 2%	++	H2	Sodium Nitrate 20 mM	+
B3	NaCl 6 % + N-N Dimethyl glycine	++	D3	Potassium chloride 5%	++	F3	Sodium Lactate 3%	++	H3	Sodium Nitrate 40 mM	+
B4	NaCl 6 % + Sarcosine	++	D4	Potassium chloride 6%	++	F4	Sodium Lactate 4%	++	H4	Sodium Nitrate 60 mM	+
B5	NaCl 6 % + Dimethyl sulphonyl propionate	++	D5	Sodium sulfate 2%	+	F5	Sodium Lactate 5%	++	H5	Sodium Nitrate 80 mM	+
B6	NaCl 6 % + MOPS	++	D6	Sodium sulfate 3%	++	F6	Sodium Lactate 6%	++	H6	Sodium Nitrate 100 mM	+
B7	NaCl 6 % + Ectoine	++	D7	Sodium sulfate 4%	++	F7	Sodium Lactate 7%	++	H7	Sodium Nitrite 10 mM	+
B8	NaCl 6 % + Choline	++	D8	Sodium sulfate 5%	++	F8	Sodium Lactate 8%	++	H8	Sodium Nitrite 20 mM	–
B9	NaCl 6 % + Phosphoryl choline	++	D9	Ethylene glycol 5%	+	F9	Sodium Lactate 9%	++	H9	Sodium Nitrite 40 mM	–
B10	NaCl 6 % + Creatine	++	D10	Ethylene glycol 10%	+	F10	Sodium Lactate 10%	++	H10	Sodium Nitrite 60 mM	–
B11	NaCl 6 % + Creatinine	++	D11	Ethylene glycol 15%	+	F11	Sodium Lactate 11%	++	H11	Sodium Nitrite 80 mM	–
B12	NaCl 6 % + L-Carnitine	++	D12	Ethylene glycol 20%	+	F12	Sodium Lactate 12%	++	H12	Sodium Nitrite 100 mM	–

*“+” and “-” indicates the ability (or not) of the strain to actively grow and reduce the redox dye present in the well, in presence of the different stresses: “++” very positive, “+” positive, “-” negative.*

### Genome Sequencing and Identification

**FIGURE 2:**
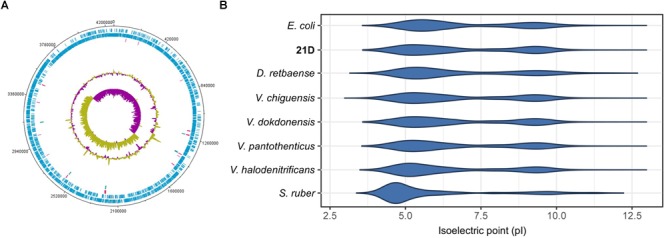
Circular map of the genome **(A)** and isoelectric point of the proteome **(B)** of *V. dokdonensis* strain 21D. In **(A)**, marked characteristics are shown from outside to the center: CDS on forward strand, CDS on reverse strand, tRNA, rRNA, GC content and GC skew. In **(B)**, are reported the isoelectric point of the proteome of *E. coli* DSM 30083, *V. pantothenticus* DSM 26^T^, *V. chiguensis* NTU-101^T^, *V. dokdonensis* Marseille-P2545, *V. halodenitrificans* JCM 12304, *S. ruber* DSM 13855, *D. retbaense* DSM 5692.

A total number of 117,330 reads were retrieved with a mean subread length of 8,461 bp and N50 of 11,849 bp. The genome showed a GC% of 36.6, with 3,944 CDS, 63 tRNA, and 18 rRNA. In Table 4 we provide detailed information related to the complete genome of *Virgibacillus* sp. strain 21D. One circular chromosome of 4,263,520bp in size was obtained after assembly (Figure 2A). Number of genes associated with general COG functional categories are reported in Table 5.

**Table 4:** Statistics of *V. dokdonensis* strain 21D genome.

Attribute	Value
Genome size (bp)	4,263,520
DNA G+C (bp)	36.6
DNA scaffolds	1
Total genes	3,944
Protein coding genes	3,761
RNA genes	86
rRNA	6, 6, 6 (5S, 16S, 23S)
tRNA	63
ncRNA	5
Pseudo genes	97
CRISPR array	1

**Table 5:** Number of genes associated with general COG functional categories.

Code	Value	%^a^	Description
J	168	4.29	Translation, ribosomal structure and biogenesis
K	288	7.36	Transcription
L	210	5.36	Replication, recombination, and repair
B	1	0.03	Chromatin structure and dynamics
D	40	1.02	Cell cycle control, Cell division, chromosome partitioning
V	63	1.61	Defense mechanisms
T	184	4.70	Signal transduction mechanisms
M	163	4.16	Cell wall/membrane biogenesis
N	62	1.58	Cell motility
U	49	1.25	Intracellular trafficking and secretion
O	109	2.78	Posttranslational modification, protein turnover, chaperones
C	183	4.67	Energy production and conversion
G	289	7.38	Carbohydrate transport and metabolism
E	288	7.36	Amino acid transport and metabolism
F	89	2.27	Nucleotide transport and metabolism
H	138	3.52	Coenzyme transport and metabolism
I	107	2.73	Lipid transport and metabolism
P	211	5.39	Inorganic ion transport and metabolism
Q	63	1.61	Secondary metabolites biosynthesis, transport, and catabolism
R	386	9.86	General function prediction only
S	307	7.84	Function unknown
–	517	13.21	Not in COGs

*^*a*^The total is based on the total number of protein coding genes in the genome.*

**FIGURE 3:**
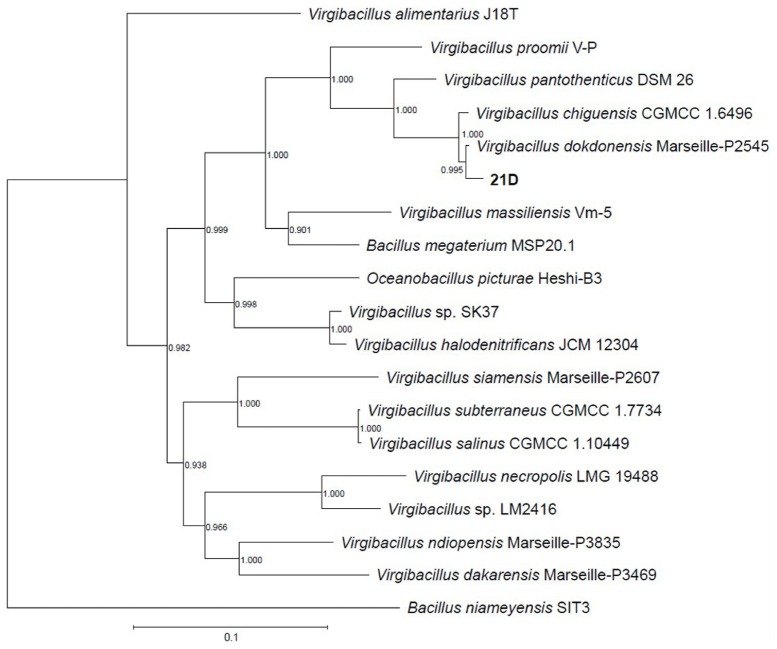
Taxonomic affiliation *V. dokdonensis* strain 21D. The phylogenetic tree was performed by using GTDB-Tk software, which applies FastTree to a concatenated alignment of 120 ubiquitous single-copy genes. Numbers at the nodes are bootstrap values obtained by repeating the analysis 100 times.

By concatenating 120 single copy genes present in the reference genomes (see accession numbers in Table 1), as well as in the genome of strain 21D (GTDB software, Parks et al., 2018) and through *in silico* DNA–DNA hybridization (dDDH, Table 6), strain 21D was phylogenetically classified as *V. dokdonensis* (Figure 3). The previous assignment (based on 800 bp-fragment of the 16S rRNA gene) of the strain 21D to the *V. pantothenticus* species as reported by De Vitis et al. (2015) was not supported. The analysis showed congruency with the taxonomic affiliation of the strain based on almost full-length 16S rRNA gene (Supplementary Figure 3).

**Table 6:** *In silico* DNA–DNA hybridization analysis by using Genome sequence-based (sub-)species delineation (GGDC) server (http://ggdc.dsmz.de/).

Reference genome (Acc. Num)	DDH	Model C.I.	Distance	Prob. DDH ≥ 70%	G+C difference
*V. alimentarius* (JFBD01)	23.2	[20.9 - 25.7%]	0.1884	0	0.52
*V. chiguensis* (FQXD01)	61.7	[58.8 - 64.5%]	0.0487	57.84	0.01
*V. dakarensis* (FUHS01)	23.9	[21.6 - 26.4%]	0.1827	0	3.12
*V. dokdonensis* (FUUZ01)	92	[89.9 - 93.7%]	0.01	96.42	0.05
*V. halodenitrificans* (CP017962)	23.2	[20.9 - 25.7%]	0.1884	0	0.83
*V. halodenitrificans* 1806 (ALEF01)	20.9	[18.7 - 23.3%]	0.2102	0	0.83
*V. halodenitrificans* (CCDO01)	21.1	[18.9 - 23.5%]	0.2081	0	12.13
*V. halodenitrificans* (FUHR01)	21.2	[19 - 23.6%]	0.2071	0	0.75
*V. halodenitrificans* JCM 12304 (BAZS01)	21.3	[19.1 - 23.8%]	0.2057	0	0.75
*V. massiliensis* (CCDP01)	20.4	[18.2 - 22.8%]	0.2155	0	0.29
*V. ndiopensis* (FZMZ01)	22.2	[20 - 24.7%]	0.1972	0	0.21
*V. necropolis* (CP022437)	25.2	[22.8 - 27.6%]	0.1729	0.01	0.69
*V. pantothenticus* (FTOS01)	25.7	[23.4 - 28.2%]	0.1689	0.01	0.64
*V. pantothenticus* (FUFM01)	26	[23.7 - 28.5%]	0.1668	0.02	0.84
*V. pantothenticus* (LGTO01)	25.7	[23.3 - 28.2%]	0.1692	0.01	0.63
*V. proomii* (FUFN01)	22.4	[20.2 - 24.9%]	0.1954	0	0.62
*V. salinus* (FNKD01)	22.6	[20.3 - 25%]	0.1941	0	0.77
*V. senegalensis* (CCXU01)	30.3	[27.9 - 32.8%]	0.1405	0.12	6.25
*V. siamensis* (FUIH01)	22.1	[19.8 - 24.5%]	0.1988	0	4.56
*V. soli* (LGPD01)	30.6	[28.2 - 33.1%]	0.1391	0.13	0.78
*V. subterraneus* (FOEH01)	22.7	[20.4 - 25.1%]	0.1933	0	0.8

### Insights Into *Virgibacillus* sp. Strain From Genomics

Automatic annotation using “Rapid Annotation using Subsystem Technology” (RAST) platform^2^ (Aziz et al., 2008; Overbeek et al., 2014; Brettin et al., 2015) showed that *V. dokdonensis* strain 21D genome contains genes that could help the strain to thrive under the osmotic stress typical of extreme environments. For example, we observed the presence of the glycine betaine transporter OpuD (A21D_00070; A21D_00685; A21D_00747; A21D_00835; A21D_02157; A21D_03789), the glycine betaine/carnitine/choline ABC transporter OpuC (A21D_01624; A21D_01625; A21D_01626; A21D_01627; A21D_03467; A21D_03468) and the glycine betaine/carnitine ABC transporter Gbu (homologous to OpuA of *B. subtilis*; A21D_02196; A21D_02672; A21D_02673; A21D_02674; A21D_02675) (Hoffmann and Bremer, 2011). Moreover, L-carnitine/gamma-butyrobetaine antiporters (A21D_02627; A21D_02815) and ectoine production CDS (A21D_03919; A21D_03920; A21D_03921) were retrieved. In particular, we compared the nucleotide sequence of *ectABC* operon of the strain 21D with that of *V. pantothenticus* DSM 26^T^, demonstrating an identity of 82% (395/480bp), 81% (1040/1284 bp), and 83% (323/387 bp) with EctA (diaminobutyric acid acetyltransferase, A21D_03921), EctB (diaminobutyric acid transaminase, A21D_03920), and EctC (ectoine synthase, A21D_03919) sequences, respectively. Finally, due to the ability of strain 21D to thrive at high MgCl_2_ concentrations, the presence of magnesium transporters was investigated and four CDS (A21D_01697, A21D_01947, A21D_02760, and A21D_02928) were unveiled from the genome annotation. The first two CDS encode for CorC protein, whereas the other CDS encode for MgtE transporters.

Taking advantage of RAST function-based comparison tool, we compared the genome of strain 21D with those of the closely related species *V. pantothenticus* DSM 26^T^, *V. chiguensis* NTU-101^T^ and *V. dokdonensis* Marseille-P2545, available on the NCBI database (Wang et al., 2008, 2015). We found the basic metabolic functions to be conserved between the four genomes. Moreover, all of them showed the presence of genes involved in osmotic stress response, while they differentiated for genes in the category “Iron acquisition and metabolism.” In *V. dokdonensis* strain 21D genome we found the presence of genes in the subcategories “siderophore metabolism” and “heme, hemin uptake and utilization systems in Gram-positives bacteria” that have not been consistently found in *V. pantothenticus, V. dokdonensis*, or *V. chiguensis* genomes. Specifically, genes related to iron uptake mediated by the siderophore petrobactin were only found in the genome of the strain 21D (in 21D: A21D_01225; A21D_01226; A21D_01227; A21D_01228, encoding for petrobactin ABC transporter components). Conversely, genes of the iron-regulated surface determinant system Isd have been only found in *V. dokdonensis* (21D and Marseille-P2545) genomes, but not in *V. pantothenticus* and *V. chiguensis* ones (in 21D: from A21D_01190 to A21D_01200; A21D_01095; A21D_01096; Table 7).

**Table 7:** Comparison among selected functions in *V. dokdonensis* 21D, *V. dokdonensis* Marseille-P2545, *V. chiguensis* NTU-101^T^, and *V. pantothenticus* DSM 26^T^ by using automatic RAST function-based comparison tool.

Role	21D	Marseille-P2545	NTU-101^T^	DSM 26^T^	CDS ID^∗^
Petrobactin ABC transporter, ATP-binding protein^a^	+	–	–	–	A21D_01228
Petrobactin ABC transporter, periplasmic binding protein^a^	+	–	–	–	A21D_01225
Petrobactin ABC transporter, permease protein I^a^	+	–	–	–	A21D_01226
Petrobactin ABC transporter, permease protein II^a^	+	–	–	–	A21D_01227
Cell surface protein IsdA, transfers heme from hemoglobin to apo-IsdC^b^	+	+	–	–	A21D_01194–A21D_01195
Heme transporter IsdDEF, lipoprotein IsdE^b^	+	+	–	–	A21D_01193–A21D_01197
Heme transporter IsdDEF, permease component IsdF^b^	+	+	–	–	A21D_01192–A21D_01198
Heme transporter analogous to IsdDEF, ATP-binding protein^b^	+	+	–	–	A21D_01191–A21D_01199
Heme-degrading monooxygenase IsdG (EC 1.14.99.3)^b^	+	+	–	–	A21D_01200
NPQTN cell wall anchored protein IsdC^b^	+	+	–	–	A21D_01196
NPQTN specific sortase B^b^	+	+	–	–	A21D_01190
Sensor histidine kinase colocalized with HrtAB transporter^b^	+	+	+	–	A21D_01096
Heme efflux system permease HrtB^b^	+	+	+	–	A21D_01095

*^*a*^In RAST subsystem: “siderophore metabolism”. ^*b*^In RAST subsystem: “heme, hemin uptake and utilization systems in Gram-positives bacteria”. ^∗^CDS numbered according to 21D automatic annotation. “+” present; “-” absence.*

Calculation of the isoelectric point (pI) of strain 21D proteome showed that the strain possesses a proteome pI different to the acid one shown by *S. ruber*, which was included in our analysis as a reference strain of the “salt-in” strategy microorganisms (Figure 2B; Oren and Mana, 2002; Oren, 2008). The pI of 21D proteome is congruent with that of *E. coli* or the halophilic strain *D. retbaense* (Spring et al., 2010). Moreover, we observed that the *Virgibacillus* strains included in our analysis showed highly similar and neutral proteome pIs (Figure 2B).

## Discussion

The ability of the strain 21D to grow in the presence of MgCl_2_ concentrations as high as 11.43–14.28% (1.2–1.5 M) is indicative of its peculiar origin of isolation, i.e., the Discovery SBI, characterized by a steep MgCl_2_ gradient ranging from the value of seawater (0.05 M) to that of the brine (5.05 M) (Hallsworth et al., 2007). Authors have previously reported the inability to isolate and grow bacterial strains from different Discovery SBI sample fractions by using media containing above 12% (1.26 M) MgCl_2_ (Hallsworth et al., 2007) and this underlines a characteristic behavior of strain 21D. Moreover, a slight (even if poor) growth of the strain was even reported at 17.14% (1.8 M) MgCl_2_. There are several examples of haloarchaeal strains that require high concentrations of magnesium for their growth in the literature, including *Halobacterium salinarum* DSM 3754^T^, *Halobaculum gomorrense* DS2807^T^, *Haloferax volcanii* DS2^T^ and *Halobaculum magnesiiphilum* MGY-184^T^ (Mullakhanbhai and Larsen, 1975; Oren et al., 1995; Grant, 2001; Shimoshige et al., 2013). For instance, authors reported that *H. magnesiiphilum* is able to grow in media with 30% (1.48M) MgCl_2_.6H_2_O (Shimoshige et al., 2013).

Three different classes of Mg^2+^ transporters have been identified in bacteria, namely CorA, MgtE, and MgtA (Groisman et al., 2013). Bacterial genomes can generally encode multiple Mg^2+^ transporters that belong to either the same or different classes. CorA and MgtE are the primary magnesium transporters in bacteria and archaea (likely being channels rather than transporters, Groisman et al., 2013) and they, indeed, show a wide phylogenetic distribution. Conversely, MgtA proteins occur only in some bacteria and are typically induced in low Mg^2+^ environments. In the genome of strain 21D, no *mgtA* genes were annotated, whereas two CDS for both CorA and MgtE were retrieved. Since Mg^2+^ plays several essential roles, e.g., acting as a cofactor in enzymatic reactions and stabilizing macromolecular complexes and membranes, the maintenance of a correct Mg^2+^ homeostasis is fundamental for cell functioning: several studies have shown that bacteria assess the levels of Mg^2+^ present in their surrounding environment or inside the cytoplasm to regulate Mg^2+^ at the required levels (Groisman et al., 2013).

Phenotypic analysis of 21D suggested a limited metabolic capability of the strain, likely underlying a narrow adaptation of the bacterium to the available C sources present in Discovery SBI. In particular, besides the simple carbon molecules of D-mannose, D-ribose, D-fructose, D-glucose, D-tagatose, and glycerol which are substrates for conserved core metabolic pathways, strain 21D showed an active metabolism on oligomers/polymers with linked α-(1→4) or α-(1→6) glycosidic bonds, i.e., dextrin, maltose, and maltotriose, and β-(1→3) or β-(1→6) glycosidic ones, specifically laminarin. Laminarin is a storage polysaccharide produced by different algal species and one of the most abundant polysaccharides present in the marine environment. It has been estimated that diatoms alone can produce about 5–15 Gt of laminarin per year, playing a pivotal role in the marine carbon cycle (Alderkamp et al., 2007). Laminarin has been detected in shallow waters, being typically associated with microalgal blooms (Unfried et al., 2018), as well as in deeper waters and sediments (Keith and Arnosti, 2001; Arnosti et al., 2005; Teske et al., 2011), but there is no literature concerning the typical depth of the Mediterranean Sea’s DHABs. This might suggest that sinking material from the upper seawater layer can reach Discovery SBI and be available to strain 21D. Furthermore, the strain showed active metabolism on pectin (a component of algal cell walls; Domozych et al., 2012), on the amino-sugar D-glucosamine, but not on chitin (a polymer present in crustacean exoskeletons). We also observed positive results for arbutin, a glycosylated hydroquinone, which is typically extracted from terrestrial plants, likely due to a broad, non-specific enzymatic activity of the strain on this substrate. Finally, strain 21D was active on inositol, a compatible solute that can be accumulated by other extremophiles (Goncalves et al., 2008), and on acetoin, a fermentation product that can be also produced by marine cognates, representing a C source for bacterial growth under unfavorable conditions (Dai et al., 2015).

Regarding carbon metabolism, we retrieved the presence of two β-glucosidases in multiple copies from the genome annotation, i.e., EC 3.2.1.86 (A21D_00222, A21D_00423, A21D_00900, A21D_02186, and A21D_02527) and EC 3.2.1.21 (A21D_00998, A21D_01912; A21D_02532), which catalyze the hydrolysis of phospho-β-D-glucosides and terminal, non-reducing β-D-glucosyl residues, respectively. As well, α-glucosidases, specifically maltose-6-phosphate α-glucosidases (EC 3.2.1.122), oligo-1.6 α-glucosidases (EC 3.2.1.10), and α-glucosidases (EC3.2.1.20), were automatically annotated, underlining the strain capability to metabolize α-linked or β-linked glycosidic bonds.

The genome of strain 21D encodes for genes that could be helpful under the osmotic stress typically encountered in DHAB extreme environments and, indeed, analysis with PM9 plates and growth experiments in the presence of osmolytes unveiled the osmoadaptation capacity of the strain. In order to adjust the cell turgor pressure, the majority of halophilic bacteria generally synthetize or uptake different osmoprotectants from the surroundings, such as ectoine, choline, carnitine and betaine (Oren, 2008). In *B. subtilis* glycine betaine has a very important role in osmoprotection, accumulating via uptake or being synthetized from the precursor choline (Hoffmann and Bremer, 2011). Specifically, three osmotically inducible transport systems have been depicted in *B. subtilis*: the ABC transporters OpuA and OpuC and the carrier OpuD, which is a member of the betaine-choline-carnitine transporter (BCCT) family. These transporters can also function for the acquisition of different osmoprotectants other than glycine betaine (Hoffmann and Bremer, 2011). Genome annotation of strain 21D revealed the presence of the abovementioned transporter systems. Interestingly, in *B. subtilis* Opu transporters have been described to contribute to glycine betaine (and other osmoprotectants such as carnitine) accumulation in cold conditions (Hoffmann and Bremer, 2011; Meadows and Wargo, 2015). Moreover, we also found the presence of the highly conserved *ectABC* gene cluster (Kuhlmann et al., 2008) responsible for ectoine production, which showed high identity with the gene cluster identified in *V. pantothenticus* DSM 26^T^. Specifically, authors underlined that the production of ectoine in DSM 26^T^ is activated when an increase of salinity or a reduction of the growth temperature occur (Kuhlmann et al., 2008). Further experiments are needed to verify and elucidate ectoine production by strain 21D in response to osmotic stress. The strain *V. halodenitrificans* PDB-F2 also showed the ability to synthesize or uptake ectoine, hydroxyectoine, trehalose, glutamic acid and betaine in presence of 12% (w/v) NaCl (Zhou et al., 2017). Although no reports are available on compatible solutes produced or acquired by strains belonging to *V. dokdonensis* species and considering our results and the available literature, we can hypothesize that this is a shared feature of *V. dokdonensis*–*V. pantothenticus* related species. Growth experiments of strain 21D with ethylene glycol and KCl showed the same trend of Biolog microarray results with good growth performances in the presence of KCl and a slight growth reduction with 20% of ethylene glycol. Interestingly, 50% ethylene glycol strongly inhibited strain 21D, whereas the high concentrations of 9 and 12% KCl did slightly influenced the strain growth.

The comparison of *Virgibacillus* spp. genome sequences suggests the ability of strain 21D to overcome the iron depletion typical of marine environments (Butler, 2005). Iron is a fundamental element for cell functioning, since it is an essential cofactor in enzymes that catalyze key processes, such as photosynthesis, respiration, and nitrate reduction (Hogle et al., 2014). Given its low concentration in seawater (Butler, 2005), marine bacteria have evolved different strategies to uptake this important element. They can, indeed, secrete or uptake siderophores (including those that are produced by other organisms), i.e., low molecular weight molecules with high affinity for insoluble Fe^3+^ ions, able to form complexes that are further absorbed by cells (de Carvalho and Fernandes, 2010). Genome analysis suggests that the strain 21D has a siderophore-mediated iron acquisition system, based on ABC transporter components and other membrane-associated proteins (de Carvalho and Fernandes, 2010). Among the bacterial genomes analyzed, only that of strain 21D possessed CDS encoding for this ability. On the other hand, genomes of both *V. dokdonensis* strains 21D and Marseille-P2545 have a gene cluster encoding for the iron-regulated surface determinant (Isd), which is one of the most studied iron uptake systems used by Gram positive bacteria to obtain iron from heme (Mazmanian et al., 2003). Heme and related molecules may be important sources of iron for marine organisms (Hogle et al., 2014). So far, some studies have investigated the abundance of iron protoporphyrin IX (heme *b*) in marine organisms and environments, i.e., phytoplankton, particulate material sampled in the North Atlantic Ocean and a mesocosm experiment set up in a Sweden fjord (Honey et al., 2013; Bellworthy et al., 2017). Specifically, heme *b* may represent 20% of the cellular iron pool of marine phytoplankton (Honey et al., 2013). Heme uptake systems have been thus searched and retrieved from genomes of marine strains (Hopkinson et al., 2008; Roe et al., 2013; Hogle et al., 2014), but the attention has been mainly devoted to the groups Proteobacteria, Bacteroidetes and Cyanobacteria, as well as a few Archaea (Hopkinson and Barbeau, 2012). No reports are available on the ability of marine Gram positive bacteria to utilize the iron contained in heme and heme-related molecules, even though this is a well-known ability exerted by Gram positive bacterial pathogens, such as *Staphylococcus aureus* (Mazmanian et al., 2003). This could suggest that in its original iron-depleted environment, the strain 21D could utilize heme to sustain its iron requirement. However, experimental verifications are needed to verify the ability of strain 21D to obtain iron both through iron-siderophore complexes and by utilizing heme.

The genus *Virgibacillus* was established in 1998 (Heyndrickx et al., 1998), while the amended description appeared in 2003 (Heyrman et al., 2003). To date, several *Virgibacillus* spp. genomes are available in the public databases, including the draft genomes of strain 21D-closely related species, i.e., *V. pantothenticus* DSM 26^T^ (Wang et al., 2015), *V. chiguensis* CGMCC 1.6496^T^ ( = NTU-101^T^) (Wang et al., 2008) and *V. dokdonensis* Marseille-P2545. Our analysis can thus contribute to enlarge the actual pool of genomic sequences available for the genus *Virgibacillus*, especially in the perspective to exploit the biotechnological potential associated with deep-sea microorganisms (Huang et al., 2018). Indeed, we have recently explored the bacterial diversity of DHABs located in the Eastern Mediterranean Sea, investigating the capability of different cultivable strains to resolve a racemic mixture of propyl ester of anti-2-oxotricyclo[2.2.1.0]heptan-7-carboxylic acid (R,S), a key intermediate for the synthesis of D-cloprostenol. Interestingly, strain 21D showed reduction of the substrate with medium/high enantioselectivity accordingly to NaCl concentration (De Vitis et al., 2015). A new transaminase was also found in strain 21D and verified to be secreted in a soluble form after the introduction of a single-mutation and the codon optimization for the expression in *E. coli* (Guidi et al., 2018).

## Conclusion

Both phenomics and genomics highlight the potential capability of *V. dokdonensis* 21D vegetative cells to adapt to the environmental conditions occurring in Discovery SBI, revealing, in particular, a high degree of consistency for C source utilization and osmoadaptation. The presence of the genetic determinants involved in siderophore-mediated iron uptake and heme utilization further suggests the ability of the strain to thrive in iron-depleted marine habitats. In conclusion, our analysis supports the evidence that cells of *V. dokdonensis* 21D are equipped with genetic and phenotypic determinants to overcome the stressful conditions of Discovery SBI, possibly avoiding their entrance in the dormancy state, which could result in spore production.

## Data Availability

The datasets generated for this study can be found in GenBank, CP018622.

## Author Contributions

EC designed the study. ZZ, RM, JB, EP, FMa, MC, and MF carried out the experiments. ZZ, EC, RM, FMo, and GM analyzed the data. EC, SB, and DD supported the research. EC wrote the manuscript. All authors contributed to manuscript revision, read, and approved the final version of the manuscript for submission.

## Conflict of Interest Statement

The authors declare that the research was conducted in the absence of any commercial or financial relationships that could be construed as a potential conflict of interest.

## Footnotes

1 https://github.com/Ecogenomics/GTDBTk/

2 http://rast.nmpdr.org/
