# Supplementary material for: Phenomics and Genomics Reveal Adaptation of Virgibacillus dokdonensis Strain 21D to Its Origin of Isolation, the Seawater-Brine Interface of the Mediterranean Sea Deep Hypersaline Anoxic Basin Discovery
Source: Front Microbiol. 2019 Jun 12;10:1304. doi: 10.3389/fmicb.2019.01304 (PMC6581673; doi:10.3389/fmicb.2019.01304)
Supplement: Supplementary file 1 [file Data_Sheet_1.docx]

Supplementary Material

**Supplementary Figure 1.** Spores of *Virgibacillus* *dokdonensis* strain 21D.


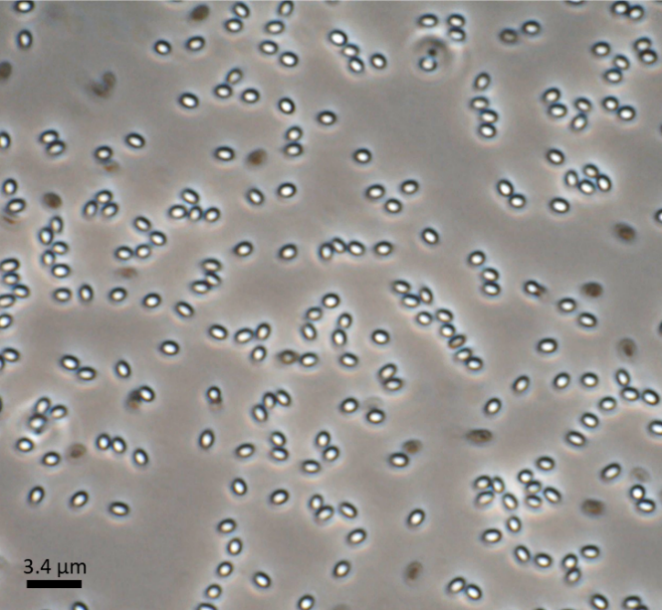


**Supplementary Figure 2 (next page).** Maximum dyeF reduction measured for *V. dokdonensis* strain 21D in the presence of different osmolytes (PM9 plate from Biolog, see Tab. 3). Values of dyeF reduction are expressed in OmniLog units (arbitrary color units due to dye reduction). In red: histograms related to the bacterial metabolic profiles in the presence of 6-10% NaCl and considered as reference. In blue: histograms of osmolytes in the presence of which the bacterium showed good metabolic performance (Class “++” or “very positive” in Tab. 3). In green: histograms related to molecules that induced a reduction of the metabolic response (Class “+” or “positive” in Tab. 3). In violet: histograms related to compounds that induced a strong reduction of metabolism (Class “-” or “negative” in Tab. 3).

**
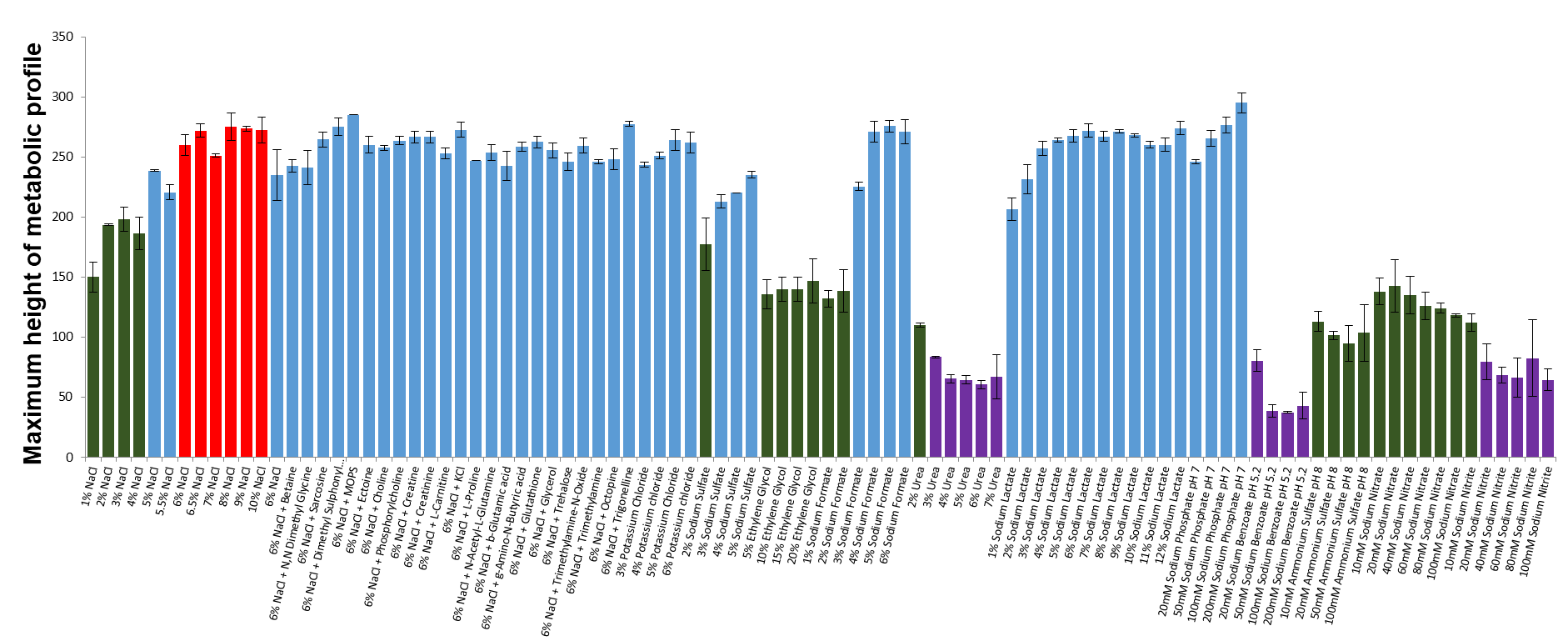
**

**Supplementary Figure 3 (next page).** Phylogenetic tree of *V.* *dokdonensis* strain 21D based on 16S rRNA gene sequence. The complete 16S rRNA sequence of *Virgibacillus* sp. strain 21D was obtained from its genomic sequence. The strains and their corresponding GenBank accession numbers for 16S rRNA genes are (type = ^T^): *Lentibacillus halophilus* PS11-2^T^ (AB191345); *Lentibacillus juripiscarius* IS40-3^T^ (AB127980); *Virgibacillus siamensis* MS3-4^T^ (AB365482); *Virgibacillus halophilus* 5B73C^T^ (AB243851); *Virgibacillus soli* CC-YMP-6^T^ (EU213011); *Virgibacillus campisalis* IDS-20^T^ (GU586225); *Virgibacillus alimentarius* J18^T^ (GU202420); *Virgibacillus oceani* MY11^T^ (KJ144820); *Virgibacillus necropolis* LMG 19488^T^ (AJ315056); *Virgibacillus carmonensis* LMG 20964^T^ (AJ316302); *Virgibacillus byunsanensis* ISL-24^T^ (FJ357159); *Virgibacillus litoralis* KCTC 13228^T^ (FJ425909); *Virgibacillus subterraneus* H57B72^T^ (FJ746573); *Virgibacillus salinus* XH22^T^ (FM205010); *Virgibacillus kekensis* YIM kkny16^T^ (AY121439); *Virgibacillus halodenitrificans* DSM 10037^T^ (AY543169); *Virgibacillus picturae* LMG 19492^T^ (AJ315060); *Virgibacillus dokdonensis* DSW-10^T^ (AY822043); *Virgibacillus chiguensis* NTU-101^T^ (EF101168); *Virgibacillus pantothenticus* IAM 11061^T^ (D16275); *Virgibacillus proomii* LMG 12370^T^ (AJ012667); *Virgibacillus xinjiangensis* KCTC 13128^T^ (DQ664543); *Virgibacillus sediminis* KCTC 13193^T^ (AY121430); *Virgibacillus kapii* KN3-8-4^T^ (LC041942); *Virgibacillus salexigens* JCM 30552^T^ (Y11603); *Virgibacillus olivae* JCM 30551^T^ (DQ139839); *Virgibacillus salarius* JCM 12946^T^ (AB197851); *Virgibacillus marismortui* KCTC 3867^T^ (AJ009793); *Virgibacillus albus* YIM 93624^T^ (JQ680032); *Virgibacillus koreensis* BH30097^T^ (AY616012); *Bacillus polygoni* YN-1^T^ (AB292819); *Bacillus aidingensis* 17-5^T^ (DQ504377); *Bacillus halochares* MSS4^T^ (AM982516); *Bacillus qingdaonensis* CM1^T^ (DQ115802); *Paenibacillus polymyxa* NCDO 1774^T^ (X60632). The evolutionary history was inferred using the Maximum Likelihood method based on the Jukes-Cantor model (Jukes et al., 1969). Evolutionary analyses were conducted in MEGA6 (Tamura et al., 2013). Numbers at the nodes are bootstrap values obtained by repeating the analysis 1000 times. The scale bar represents a 1% nucleotides sequence divergence.


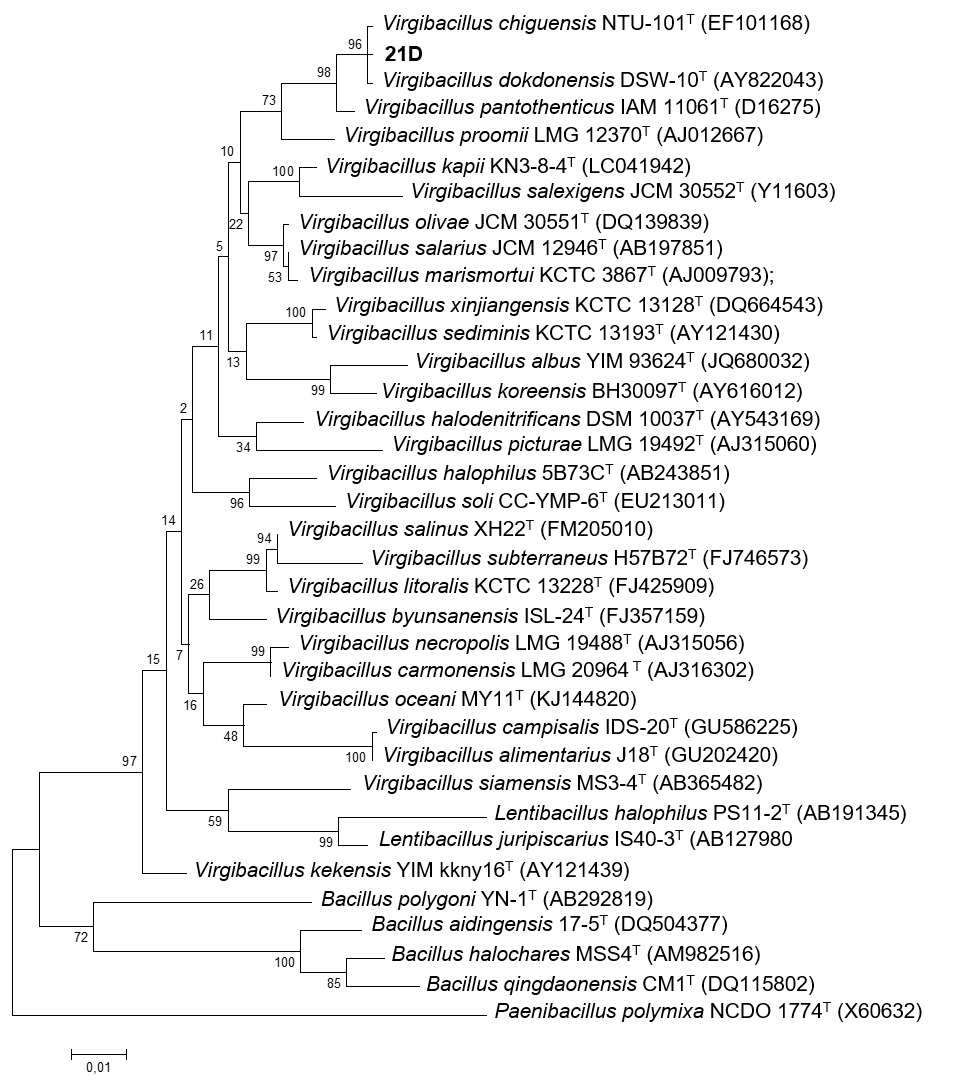


**References**

Jukes, T.H., Cantor, C.R. (1969) Evolution of protein molecules. In Munro HN, editor, Mammalian Protein Metabolism; pp. 21-132, Academic Press, New York.

Tamura, K., Stecher, G., Peterson, D., Filipski, A., Kumar, S. (2013) MEGA6: Molecular Evolutionary Genetics Analysis version 6.0. *Mol Biol Evol*. 30(12), 2725–9. doi: 10.1093/molbev/mst197.
